# Angiopoietin 1 influences ischemic reperfusion renal injury via modulating endothelium survival and regeneration

**DOI:** 10.1186/s10020-019-0072-7

**Published:** 2019-02-13

**Authors:** Wen-Chih Chiang, Yu-Chin Huang, Ten-I Fu, Ping-Min Chen, Fan-Chi Chang, Chun-Fu Lai, Vin-Cent Wu, Shuei-Liong Lin, Yung-Ming Chen

**Affiliations:** 1 Department of Internal Medicine, National Taiwan University Hospital, No. 7, Jhong-Shan South Road, Taipei, 100 Taiwan; 2 Department of Internal Medicine, Taoyuan General Hospital, Ministry of Health and Welfare, Taoyuan City, Taiwan; 3 Graduate Institute of Physiology, College of Medicine, National Taiwan University, Taipei, Taiwan; 4 College of Medicine, National Taiwan University, Taipei, Taiwan

**Keywords:** Acute kidney injury, Angiopoietin 1, Endothelium, Ischemic reperfusion injury, Kidney

## Abstract

**Background:**

Damage to the endothelium due to ischemia reperfusion injury (IRI) leads to a disruption of the microvasculature, which could be influenced by angiopoietin 1 via its effects on endothelium. We investigated the physiological and therapeutic roles of angiopoietin 1 in renal IRI using *angiopoietin 1* knockout and over-expression mice.

**Methods:**

Renal IRI was induced by clamping the right renal artery seven days after left uninephrectomy for 25 min followed by reperfusion. A whole body *angiopoietin 1* knockout was achieved by induction with tamoxifen. The renal tubule over-expression of *angiopoietin 1* was induced by doxycycline.

**Results:**

In the normal mice, the renal expression of *angiopoietin 1* increased 7 days to 14 days after IRI. The *angiopoietin 1* knockout caused a delay in the recovery of renal function, less tubular regeneration and more residual tubular necrosis. The endothelial density was lower and the VE-cadherin protein loss was greater in the knockout mice. The over-expression of *angiopoietin 1* attenuated the tubular necrosis and renal function impairment 1 and 3 days after IRI. The loss of the endothelium was ameliorated in the over-expression mice. This protective effect was associated with the up-regulation of the gene expression of *epidermal growth factor*, *hepatocyte growth factor,* and *insulin like growth factor-1* and less tubular apoptosis. The over-expression of *angiopoietin 1* stimulated *tumor necrosis factor-α*, *C-C chemokine receptor type 2 and CX3C chemokine receptor 1* inflammatory gene expression, but did not influence macrophage infiltration.

**Conclusions:**

Altogether, the augmentation and downregulation of angiopoietin 1 attenuated renal damage and impaired renal recovery, respectively, by influencing the survival/regeneration of the endothelium. The manipulation of angiopoietin 1 represents a novel therapeutic approach for the treatment of ischemic kidney injury.

**Electronic supplementary material:**

The online version of this article (10.1186/s10020-019-0072-7) contains supplementary material, which is available to authorized users.

## Background

Acute kidney injury (AKI) occurs in a variety of clinical settings and is usually associated with excessively high morbidity and mortality. The renal injury status and recovery process can determine the outcome of patients with AKI and influence the development and progression of chronic kidney disease (Wu et al., 2014; Wu et al., 2011). Clinically, most cases of AKI are caused by ischemic reperfusion injury (IRI) during/after sepsis or surgery. Therefore, understanding the pathophysiology of ischemic reperfusion renal injury is highly important for developing novel strategies for the treatment of ischemic AKI.

The initiating event of acute ischemic reperfusion injury is an abrupt decrease in renal blood flow. However, a full recovery of renal microvascular blood flow after acute ischemia occurs in only one-third to one-quarter of cases (Conger et al., 1995). The sustained decrease in blood flow after an acute insult keeps the renal tissues persistently ischemic and aggravates renal damage. Several mechanisms can lead to a decrease in renal blood flow after ischemia. First, the endothelium dies and sloughs from the basement membrane after IRI. A certain degree of functional impairment is observed in the blood vessel with a poor response to endothelium-dependent vasodilators (Conger et al., 1995), such as nitric oxide (Kwon et al., 2009) and acetylcholine (Lieberthal et al., 1989). In addition, a blood vessel without an endothelium is prone to prolonged vasoconstriction and sporadic cessation of blood flow in peritubular capillaries, which usually occurs during the reperfusion phase (Brodsky et al., 2002; Yamamoto et al., 2002). In addition to the reduced total renal blood flow, a regional alteration in renal blood flow occurs during AKI, and the perfusion of outer medulla is reduced disproportionately more than the total kidney (Le Dorze et al., 2009; Mason et al., 1984; Karlberg et al., 1983). Damage to renal vascular endothelial cells plays a key role in the continued ischemia of the renal tubular epithelium and inflammatory response observed during ischemic AKI (Sutton et al., 2002). Actin cytoskeleton and junctional alterations also occur in renal microvascular endothelial cells during injury (Sutton et al., 2003). In the cerebral and coronary vasculature, the separation of the endothelial tight junctions, loss of endothelial attachment to the basement membrane, endothelial blebbing, and endothelial necrosis were observed following ischemic injury (Pomfy et al., 1995; van der Poll & van Deventer, 1999). Increased renal peritubular capillary permeability was found to be a consequence of ischemic AKI in animal models (Sutton et al., 2003; Olof et al., 1990). These events lead to fluid leakage into the pericapillary space resulting in the formation of interstitial edema and reduction of renal blood flow. Therefore, increasing renal blood flow and protecting the endothelium from damage may attenuate ischemic reperfusion renal injury. Indeed, the administration of differentiated endothelial cells has been shown to ameliorate the no-reflow phenomenon in the post-ischemic kidney and result in significant functional protection in IRI (Brodsky et al., 2002).

The angiopoietin system is a well-known system involved in angiogenesis. The angiopoietins and their cognate receptors, mainly tyrosine kinase with Ig and EGF homology domains 2 (Tie 2), are involved in vascular generation, development and maturation (Morisada et al., 2006; Lobov et al., 2002; Maisonpierre et al., 1997; Suri et al., 1996). Angiopoietin 1 (Angpt1) is generated from vascular mural cells, pericytes, and certain other cells (Maisonpierre et al., 1997; Suri et al., 1996). Angpt1 participates in vascular differentiation through angiogenesis, which is the process of the growth and remodelling of existing vessels. Angpt1 is also involved in the maintenance and turnover of blood vessels in mature animals. Angpt1 oligomers and multimers bind to Tie 2, leading to enhanced endothelial survival and endothelial cell–cell stabilization (Kim et al., 2005). These Angpt1 effects may play a protective role in acute renal IRI. However, certain clinical conditions may suppress Angpt1 expression and influence the development of or recovery from IRI. For example, circulatory Angpt1 is suppressed in patients with severe sepsis or acute respiratory distress syndrome (Giuliano Jr. et al., 2007; Ricciuto et al., 2011). In animal studies, the expression levels of Angpt1 in the diaphragm, liver, and lung were significantly attenuated following lipopolysaccharide administration in mice (Mofarrahi et al., 2008). Furthermore, plasma Angpt1 is associated with a lower risk of AKI in human beings (Robinson-Cohen et al., 2016). Among septic shock patients, the Angpt1 level in the AKI group was significantly lower than that in the non-AKI group (Ebihara et al., 2016). In an animal model, the administration of Angpt1-treated early endothelial outgrowth cells can reduce renal damage in acute ischemic kidney injury (Patschan et al., 2013).

To further explore the role of Angpt1 in the evolution of ischemic AKI in vivo, in this study, we used conditional *Angpt1* knockout mice to mimic the clinical condition of suppressed Angpt1 and inducible *Angpt1* over-expression mice that had enhanced renal Angpt1 expression. According to our data, Angpt1 plays an important role in endothelium survival and recovery from renal IRI, suggesting that the augmentation of Angpt1 could represent a new therapeutic strategy for preventing and managing AKI.

## Methods

### Construction of inducible Angpt1 knockout and over-expression mice

**Fig. 1:**
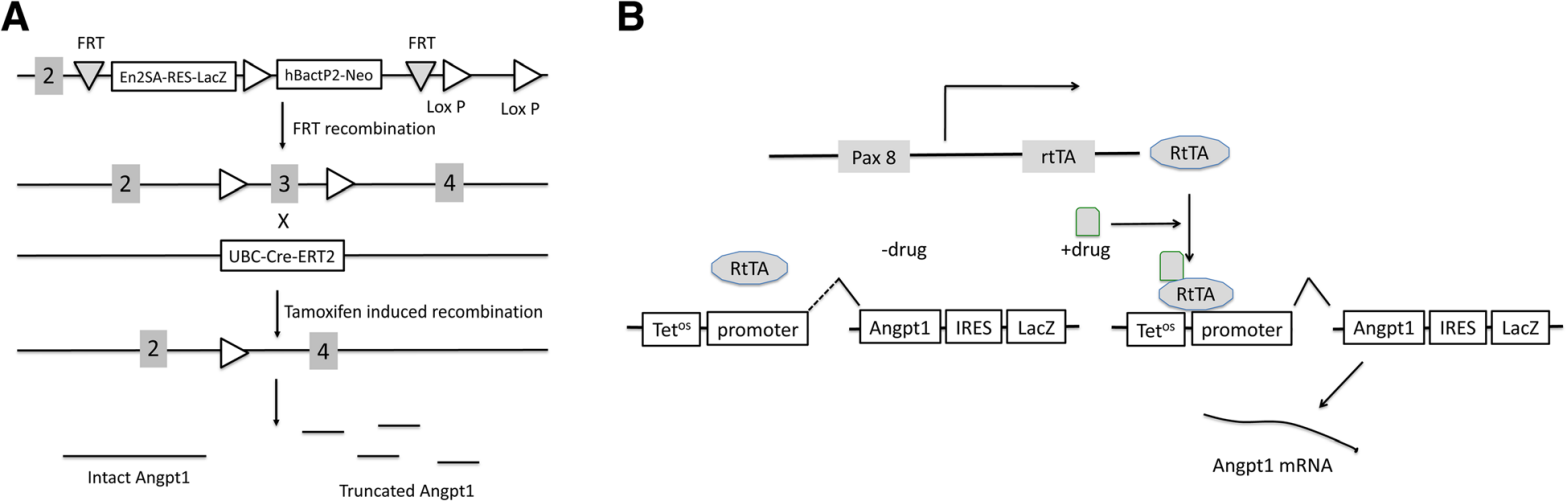
**a** Design of inducible whole body *Angpt1* knockout mice. *Angpt1* exon 3 floxed Angpt1^flox/flox^ mice were mated with UBC/CreErt2(Tg) mice to generate the UBC-CreErt2(Tg)/Angpt1^flox/flox^ mice. After the induction with tamoxifen, exon 3 of *Angpt1* was excised. The truncated *Angpt1* mRNA was expressed and degraded or abnormal Angpt1 protein was translated. **b** Design of renal tubule specific inducible *Angpt1* over-expression mice. Pax8-rtTA(Tg) mice were mated with pTre-hAngpt1(Tg) mice to generate the Pax8-rtTA(Tg)/pTre-hAngpt1(Tg) mice. After the induction with doxycycline, the renal tubule began to generate human *Angpt1* mRNA

To generate the inducible *Angpt1* knockout mice, LoxP flanked *Angpt1* exon 3 (Angpt1^flox/flox^) mice were generated at the National Taiwan University Research Center for Medical Excellence - Division of Genomic Medicine, Transgenic Core. An embryonic stem cell clone carrying loxP flanked *Angpt1* exon 3 DNA (Fig. 1a) was purchased from Sanger (UK). After the flipase recombinase target recombination in the embryonic cells, the Lac Z/neo cassette was excised, and exon 3 was flanked with two loxP sequences that were susceptible to Cre recombinase excision. The Angpt1^flox/flox^ mice were mated with UBC-CreErt2(Tg) mice (Jackson Laboratory stock #008083) to generate the UBC-Ert2Cre(Tg)/Angpt1^flox/flox^ mice. The UBC-CreErt2(Tg)/Angpt1^flox/flox^ mice express estrogen receptor fusion Cre recombinase in all cells. After the induction with tamoxifen (1 mg/mouse for 5 days/week for 2 successive weeks) in the 8~10 weeks old UBC-CreErt2(Tg)/Angpt1^flox/flox^ mice mice, the *Angpt1* exon 3 was excised, abnormal *Angpt1* mRNA was generated and then degraded or abnormal Angpt1 protein was generated. After the *Angpt1* knockout, the genotype was confirmed by PCR diagnostic tests using suitable primers (Table 1). The littermate Angpt1^flox/flox^ mice were used as the control mice of UBC-CreErt2(Tg)/Angpt1^flox/flox^ mice. They received tamoxifen treatment the same as the experimental *Angpt1* knockout mice. The Tet-on system *Angpt1* over-expression mice were generated by mating Pax8-rtTA(Tg) mice (provided by Dr. Robert Koesters, University of Heidelberg) with pTre-hAngpt1(Tg) mice (provided by Dr. Daniel Dumont, University of Toronto) to generate Pax8-rtTA(Tg)/pTre-hAngpt1(Tg) mice (Fig. 1b). The pTre-hAngpt1(Tg) mice were generated by by utilizing a pTRE LacZ/Ang1 construct to generate transgenic mice. In the presence of doxycycline (100 μg/mL in 5% glucose drinking water), rtTA expressed by Pax8-rtTA(Tg) cDAN binds to tetracycline-response operon promoter element and initiates transcription from the cytomegalovirus (CMV) promoter of the LacZ/hAngpt-1 cDNA. Expression of human Angpt1 was induced by doxycycline 7 days before uninephrectomy in the 8~10 weeks old mice until sacrifice (total doxycycline administration duration: 14~21 days). The littermate pTre-hAngpt1(Tg) mice were used as the control mice of Pax8-rtTA(Tg)/pTre-hAngpt1(Tg) human *Angpt1* over-expression mice. They received doxycycline treatment the same as the Pax8-rtTA(Tg)/pTre-hAngpt1(Tg) human *Angpt1* over-expression mice. The genotype of the Pax8-rtTA(Tg)/pTre-hAngpt1(Tg) mice was confirmed by PCR using suitable primers (Table 1). We use this type of transgenic mice to express *Angpt1* in renal tubule and exert its paracrine function on renal endothelium.

**Table 1:** Primers

Primers	Sequence
Primers used in Genotyping PCR reaction	
Angpt1 knock out F	5’ AAC CTT TCC CTC CCC TTT TT 3’
Angpt1 knock out R	5’ TTG GTG CTG CTG AAG AAA TG 3’
UBC-CreErt2 F	5’-GCA AGA ACC TGA TGG ACA-3’
UBC-CreErt2 R	5’- CTA GAG CCT GTT TTG CAC-3’
Pax8-rtTA F	5’-CCA TGT CTA GAC TGG ACA AGA-3’
Pax8-rtTA R	5’-CTC CAG GCC ACA TAT GAT TAG-3’
rtTA-hAngpt1 F	5’ ATA GGA ACC AGC CTC CTC TCT 3’
rtTA-hAngpt1 R	5’ AAG GAC ACT GTT GTT GGT GGT 3’
Primers used in quantitative RT-PCR	
Mouse GAPDH F	5’ CCT GGA GAA ACC TGC CAA GTA T 3’
Mouse GAPDH R	5’ CAT ACC AGG AAA TGA GCT TGA CA 3’
Human Angpt1 F	5’ TCA ATC TTT GCA CTA AAG AAG GTG T 3’
Human Angpt1 R	5’ GTC CAA CCT CCC CCA TTG AC 3’
Mouse Angpt1 F	5’ GGC CAC CAT GCT TGA GAT AG 3’
Mouse Angpt1 R	5’ TTT CAA GTC GGG ATG TTT GA 3’
Mouse TNF-α F	5’ TAG CCA GGA GGG AGA ACA GA 3’
Mouse TNF-α R	5’ TTT TCT GGA GGG AGA TGT GG 3’
Mouse IL-1β F	5’ CCC AAG CAA TAC CCA AAG AA 3’
Mouse IL-1β R	5’ GCT TGT GCT CTG CTT GTG AG 3’
Mouse MCP-1 F	5’ AGC ACC AGC CAA CTC TCA CT 3’
Mouse MCP-1 R	5’ CGT TAA CTG CAT CTG GCT GA 3’
Mouse IL-6 F	5’ AGA CAA AGC CAG AGT CCT TCA 3’
Mouse IL-6 R	5’ GGT CCT TAG CCA CTC CTT CTG 3’
Mouse CX3CR1 F	5’ GGA GAC TGG AGC CAA CAG AG 3’
Mouse CX3CR1 R	5’ CCT GAT CCA GGG AAT GCT AA 3’
Mouse CXCL2 F	5’ CAG ACT CCA GCC ACA CTT CA 3’
Mouse CXCL2 R	5’ CAG TTC ACT GGC CAC AAC AG 3’
Mouse CCL3 F	5’ ATG AAG GTC TCC ACC ACT GC 3’
Mouse CCL3 R	5’ GAT GAA TTG GCG TGG AAT CT 3’
Mouse CCR2 F	5’ ATT CTC CAC ACC CTG TTT CG 3’
Mouse CCR2 R	5’ GAT TCC TGG AAG GTG GTC AA 3’
Mouse CCL17 F	5’ AGT GGA GTG TTC CAG GGA TG 3’
Mouse CCL17 R	5’ CTG GTC ACA GGC CGT TTT AT 3’
Mouse Arginase 1 F	5’ AGC ACT GAG GAA AGC TGG TC 3’
Mouse Arginase 1R	5’ CAG ACC GTG GGT TCT TCA CA 3’
Mouse IGF-1 F	5’ GGC ATT GTG GAT GAG TGT TG 3’
Mouse IGF-1 R	5’ TCT CCT TTG CAG CTT CGT TT 3’
Mouse EGF F	5’ AGT CAG ACC GAA AGA GCT GTG 3’
Mouse EGF R	5’ CCT GGG AAT TTG CAA ACA GT 3’
Mouse HGF F	5’ CGC TAC GAA GTC TGT GAC ATT C 3’
Mouse HGF R	5’ CAT GGG ACC TCT GTA GCT TTC AC 3’
Mouse PDGF-A F	5’ GAT GAG GAC CTG GGC TTG 3’
Mouse PDGF-A R	5’ GAT CAA CTC CCG GGG TAT CT 3’
Mouse PDGF-B F	5’ CCC ACA GTG GCT TTT CAT TT 3’
Mouse PDGF-B R	5’ GTG AAC GTA GGG GAA GTG GA 3’

### Animal model: Ischemic reperfusion renal injury model

The C57BL6/J (8~10 weeks old), UBC-CreErt2(Tg)/Angpt1^flox/flox^ (11~13 weeks old, 1 week after the completion of 2 week of tamoxifen induction) and Pax8-rtTA(Tg)/pTre-hAngpt1(Tg) (10~12 weeks old, 1 week after the initiation of doxycycline induction) mice were subjected to right uninephrectomy. One week after uninephrectomy, the ischemic reperfusion renal injury was achieved by clamping the left renal artery for 25 min, followed by reperfusion of renal blood flow by removing the blood vessel clamp. The ischemia was confirmed by red black colour changes after the renal artery clamping, and reperfusion was confirmed by a regaining of the fresh red colour after removing the vessel clamper. The sham mice received right uninephrectomy and abdominal wall open operation but without ischemic reperfusion renal injury. All procedures were performed at a body temperature of 37 °C.

### Renal histopathology, immunofluorescence staining and immunohistochemistry

The kidneys were not fixed or fixed in paraformaldehyde and sectioned into 6-μm slices. For the morphological examination, the kidney slices were stained with periodic acid-Schiff and haematoxylin. The classification of healthy, injured, necrotic or recovering tubules was defined according to the morphology, nuclear number and integrity of renal tubules. Healthy tubule shows a normal cellular morphology. Necrotic tubules exhibit a compromised monolayer with evident cell loss and disappearance of cell morphology. Injured tubules exhibit a thinned cellular monolayer and contain fewer nuclei. Recovering tubules exhibit a more normal cellular morphology and a similar number of nuclei to healthy tubules (Hesketh et al., 2014). For the immunostaining, the kidney slices were subjected to antigen retrieval with microwave treatment in a sodium citrate buffer. The tissue sections were permeabilized with 0.3% Triton-X 100 in PBS, blocked with 5% normal goat or donkey serum, and probed with the primary antibody. After washing, the tissue sections were probed with fluorophore-conjugated secondary antibodies (Invitrogen, Waltham, MA, USA). Then, the tissue sections were mounted and fluorescence microscopy was performed. In the immunohistochemical study, a peroxidase-conjugated linker was added after washing off the primary antibody. The staining was performed using DAB as a substrate, and the nucleus was counterstained with haematoxylin. The CD31 density (vessel density) index was calculated by dividing the CD31-positively stained area by the kidney area. The tubular apoptosis was performed with ApopTag Peroxidase In Situ Apoptosis Detection Kit (Merk Millipore, Darmstadt, Germany) The amount of Ki67, CD68 or apoptotic tubular cells in the cortical medulla junction were counted by semi-quantitative method per high power filed under microscopy. Eight to twelve 400x magnification fields were taken from each mouse. The average numbers were taken into the calculation of the means for each group.

### Gene expression and quantitative PCR, Western blotting, and enzyme linked immunosorbent assay

The gene expression levels were evaluated using the real-time quantitative PCR method. RNA was extracted from the isolated kidney, subjected to DNase digestion (Qiagen, Valencia, CA, USA), and reverse-transcribed using oligo-dT primers (Tools Quant II fast RT kit, BioTools, Lognholme, Australia). The gene expression levels were determined by real-time quantitative PCR using Sybr green. Inflammatory and growth factor genes, such as tumour necrosis-α (*TNF-α), interleukin-1β (IL-1β), interleukin-6, (IL-6), monocyte chemoattractant protein-1 (MCP-1), CX3C chemokine receptor 1 (CX3CR1), Chemokine (C-X-C motif) ligand 2 (CXCL2), Chemokine (C-C motif) ligand 3 (CCL3), Chemokine (C-C motif) ligand 17 (CCL17),* C-C chemokine receptor type 2(*CCR2), arginase 1 (Arg1), platelet derived growth factor-A (PDGF-A), platelet derived growth factor-B (PDGF-B), insulin like growth factor-1 (IGF-1), epidermal growth factor (EGF), and hepatocyte growth factor (HGF)* were analysed using suitable primers (Table 1). The gene expression levels in each mouse were analysed using the relative quantitative method. The *Glyceraldehyde 3-phosphate dehydrogenase* gene was used as an internal control.

The kidney tissue was lysed with RIPA buffer containing proteinase and phosphatase inhibitors. The protein was subjected to SDS-PAGE electrophoresis, transferred to a PVDF membrane, and blotted with the suitable primary antibody. Human Angpt1 was measured with enzyme linked immunosorbent assay (ELISA) kit for human Angpt1 (R&D, Minneapolis, USA). The measure human Angpt1 protein concentration was corrected by the sampled tissue protein concentration after measurement with ELISA.

### Materials

The anti-CD31 antibody was obtained from eBioscience (San Diego, CA, UA). The anti-VE-cadherin and anti-CD68 antibody were obtained from Abcam (Cambridge, MA, USA). The anti-Ki67 antibody was purchased from Merck Millipore (Burlington, MA, USA). Anti-β-actin was obtained from Millipore Sigma (Burlington, MA, USA). The antibody against mouse Angpt1 antibody was from Gene Tex Inc. (San Antonio, Tx, USA).

### Biochemistry, various measurements and statistics

The blood urea nitrogen and serum/urine creatinine levels were determined by the central lab of National Taiwan University Hospital. The urine albumin level was evaluated using ELISA kit (Bethyl Laboratory, Montgomery, TE, USA). The degree of proteinuria was evaluated according to the urine albumin/creatinine ratio. All values in the bar graphs were expressed as mean ± standard error. Data between groups were analysed with Mann-Whitney U test or Bonferroni test.

## Results

### Renal Angpt1 expression was up-regulated after IRI. Angpt1 deficiency impaired the renal recovery after IRI

**Fig. 2:**
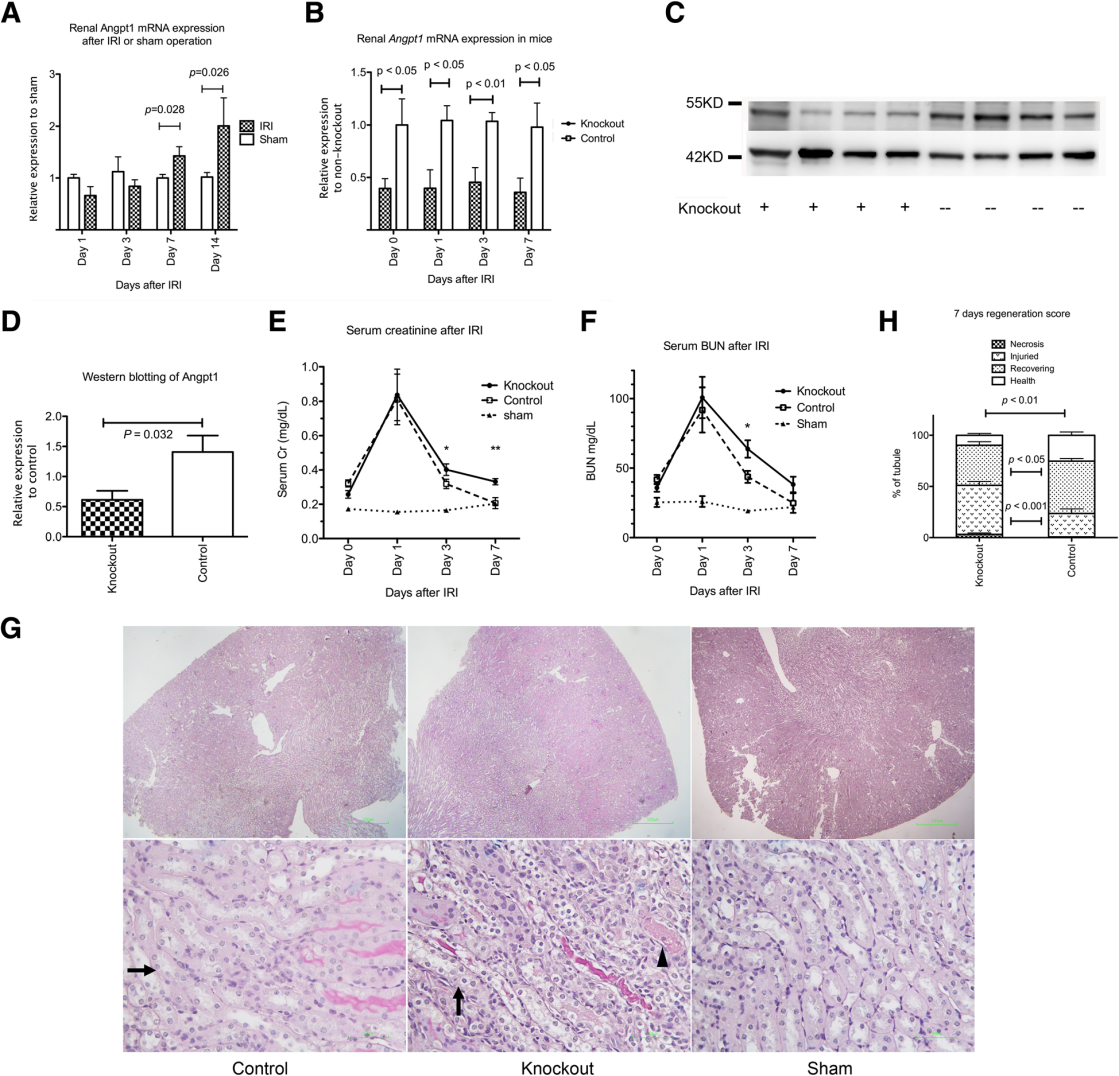
**a** Expression of *Angpt1* mRNA 1, 3, 7, and 14 days after IRI in wild mice. The expression of *Angpt1* increased 7 days and 14 days after IRI. (*N* = 6 per sham and IRI groups at each time point) **b** The expression of *Angpt1* in kidney was significantly suppressed in the *Angpt1* knockout mice on the day of IRI and 1, 3, or 7 days after IRI compared to that in the control mice (*N* = 6, 6, 12, and 17 in knockout group on day 0, 1, 3, and 7 days respectively, *N* = 6, 6, 12, and 15 in control group on day 0, 1, 3, and 7 days respectively, *P* < 0.05 or *P* < 0.01) **c** & **d** Western blotting of mouse Angpt1 3 days after IRI. The Angpt1 protein level was lower in the knockout than control mice. (*N* = 5 for each group, *P* < 0.05) **e** Serum creatinine level was higher in the *Angpt1* knockout mice 3 and 7 days after IRI. (*N* = 17 in knockout group, *N* = 15 in control group, *N* = 5 in the sham group, *P < 0.05, **P < 0.01) **f** Serum BUN level was higher in *Angpt1* knockout mice 3 days after IRI. (*N* = 17 in knockout group, *N* = 15 in control group, *N* = 5 in the sham group, *P < 0.05) **g** PAS staining of renal tissue in control, knockout and sham mice. The remaining tubular necrosis was more severe and the renal tubular recovery was less in the knockout mice 7 days after IRI. Arrow indicates recovery tubule. Arrow head indicates necrotic tubule. **h** Semi-quantitative analysis of the regeneration score in the control and knockout mice. The recovery percentage was lower and the necrosis percentage was higher in the *Angpt1* knockout mice 7 days after IRI (*N* = 17 in the knockout group, *N* = 15 in the control group)

Neoangiogenesis is an important repair process after renal IRI. The mRNA expression of *Angpt1* in the kidney began to increase and remained high 7 and 14 days after IRI (Fig. 2a). To determine whether the up-regulation of *Angpt1* plays a physiological role after renal IRI, we generated inducible whole-body *Angpt1* knockout mice. Compared to the control mice, the mRNA expression of intact *Angpt1* in the kidney was significantly suppressed in these knockout mice on the day of IRI, and 1, 3 and 7 days after IRI (Fig. 2b). Renal protein level of Angpt1 was also lower in the knockout mice than control mice 3 day after IRI (Fig. 2c & d) *Angpt1* knockout in the adulthood did not significantly change daily activity or gross organ appearance of the mice. On day 0 before the IRI, there was no difference of renal function between knockout and control mice (Additional file 1: Figure S1A and 1B). The glomerular permeability, which was assessed according to the degree of urine albuminuria to creatinine ratio, was also not influenced by the *Angpt1* knockout on the day of IRI. (Additional file 1: Figure S1C) The renal pathology also did not reveal any significant anomaly immediately before IRI in the knockout mice. Thus, the effect of the *Angpt1* knockout on renal IRI is not due to the effect of the *Angpt1* knockout during the period between the tamoxifen induction and IRI. The *Angpt1* knockout did not significantly influence renal function 1 day after IRI. However, renal function recovery 3 and 7 days after IRI was significantly delayed in the knockout mice (Fig. 2e and f). The remaining necrosis was more prominent and renal tubular recovery was significantly impaired in the *Angpt1* knockout mice 7 days after IRI (Fig. 2g) according to the regeneration score evaluated 7 days after IRI (Fig. 2h).

### Decreases in Angpt1 impaired renal microvascular regeneration and integrity after IRI

**Fig. 3:**
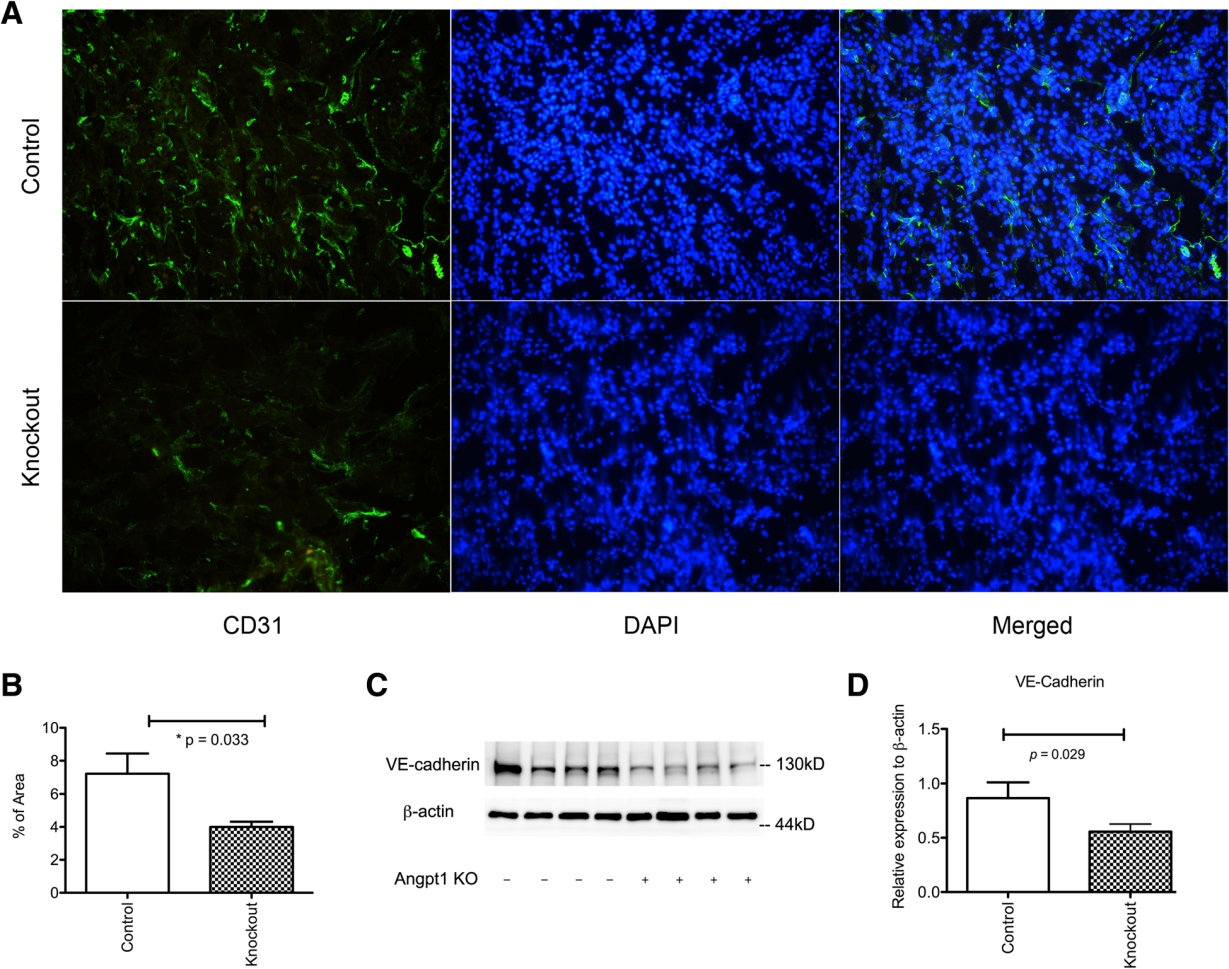
**a** & **b** CD31 endothelial staining and quantitative analysis of CD31 positive area 3 days after IRI in the control and knockout mice. The CD31 staining was less pronounced in the *Angpt1* knockout mice. (*N* = 9 in the control group, *N* = 12 in the knockout group) **c** & **d** VE-cadherin protein expression 3 days after IRI in the control and knockout mice. The protein expression of VE-cadherin was lower in the *Angpt1* knockout mice (*N* = 4 per group)

During the repair process after renal IRI, neoangiogenesis occurs to re-establish the microcirculation that is lost during ischemic reperfusion injury. The knockout process prevented the up-regulation of intact Angpt1 and impaired neoangiogenesis after IRI as revealed by a decreased endothelial CD31 density in the kidney 3 days after IRI (Fig. 3a and b) Angpt1 is also an important factor for maintaining the integrity and permeability of the endothelium. Thus, the expression of the intercellular adhesion molecule protein VE cadherin was lower in the Angpt1 knockout mice 3 days after IRI (Fig. 3c, d).

### Angpt1 deficiency did not change the inflammatory process but impaired tubular regeneration after IRI

**Fig. 4:**
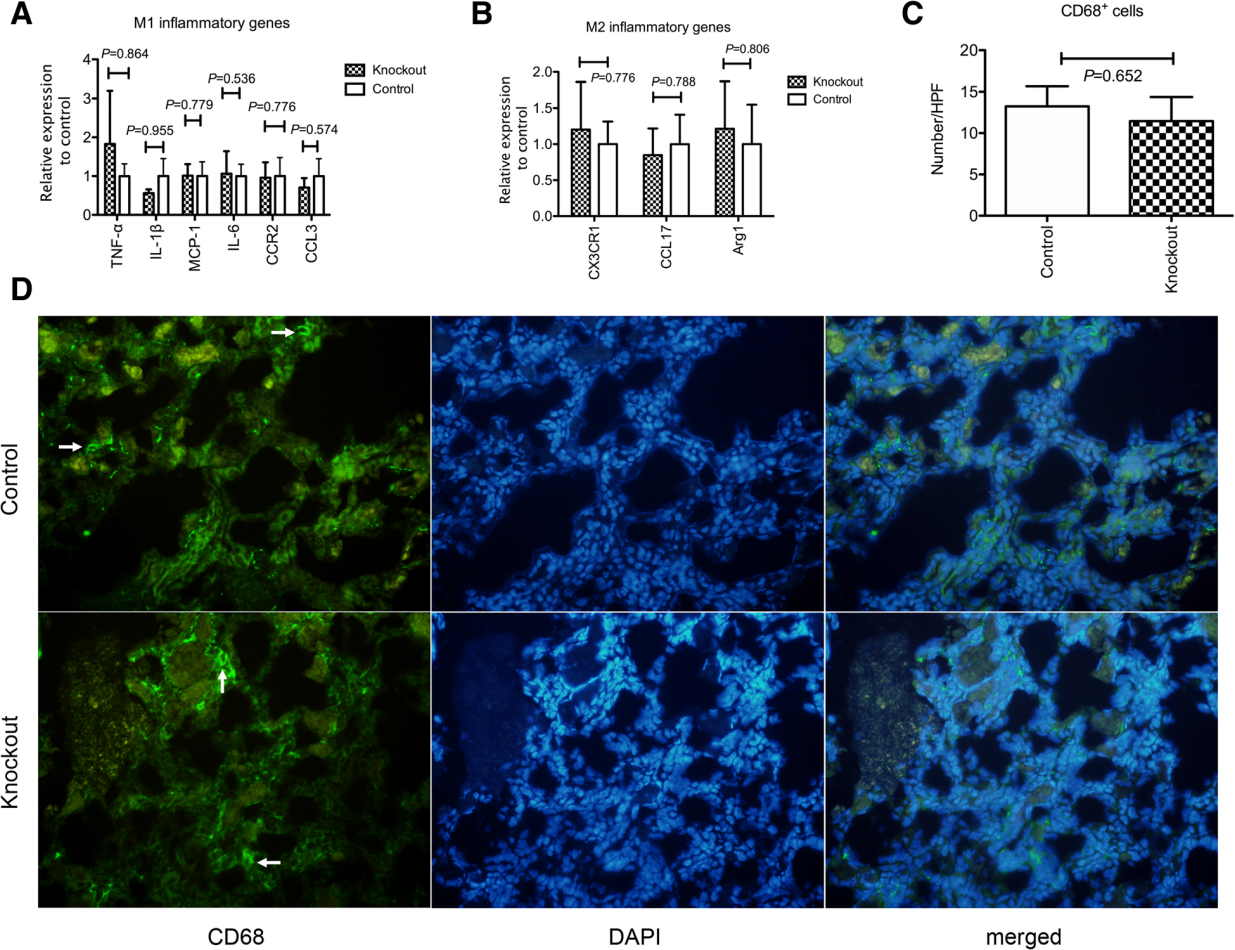
**a** M1 inflammatory genes expression 3 days after IRI in the whole kidney. There was no difference of *TNF-α, IL-1β, MCP-1, IL-6, CCR2* and *CCL3* genes expression between the *Angpt1* knockout and control mice. (*N* = 9 per group) **b** M2 inflammatory genes expression 3 days after IRI in the whole kidney. There was no difference of *CX3CR1, CCL17 and Arginase 1* genes expression between the *Angpt1* knockout and control mice. (*N* = 6 per group) **c** & **d** CD68 positive cells infiltration 3 days after IRI in the kidney. There was no difference of the amount of CD68 positive cells between the *Angpt1* knockout and control mice. Arrow indicates CD68 positive cells (*N* = 6 per group)

Inflammatory reactions play an important role in ischemic reperfusion injury. Angpt1 has been shown to influence inflammation. However, in our study, we did not observe a difference in the expression of M1 inflammatory genes *TNF-α, IL1-β, MCP-1, CCR2, CCL3* and *IL-6* (Fig. 4a) or M2 inflammatory genes *CX3CR1, CCL17, and Arg1* (Fig. 4b) expression in the kidney 3 days after IRI between knockout and control mice. The above genes expression was also not different 7 days after ischemic injury (Additional file 2: Figure S2). Macrophage infiltration also did not differ between these two groups 3 days after IRI (Fig. 4c, d). Thus, the *Angpt1* knockout did not influence the above-mentioned inflammatory reactions.

**Fig. 5:**
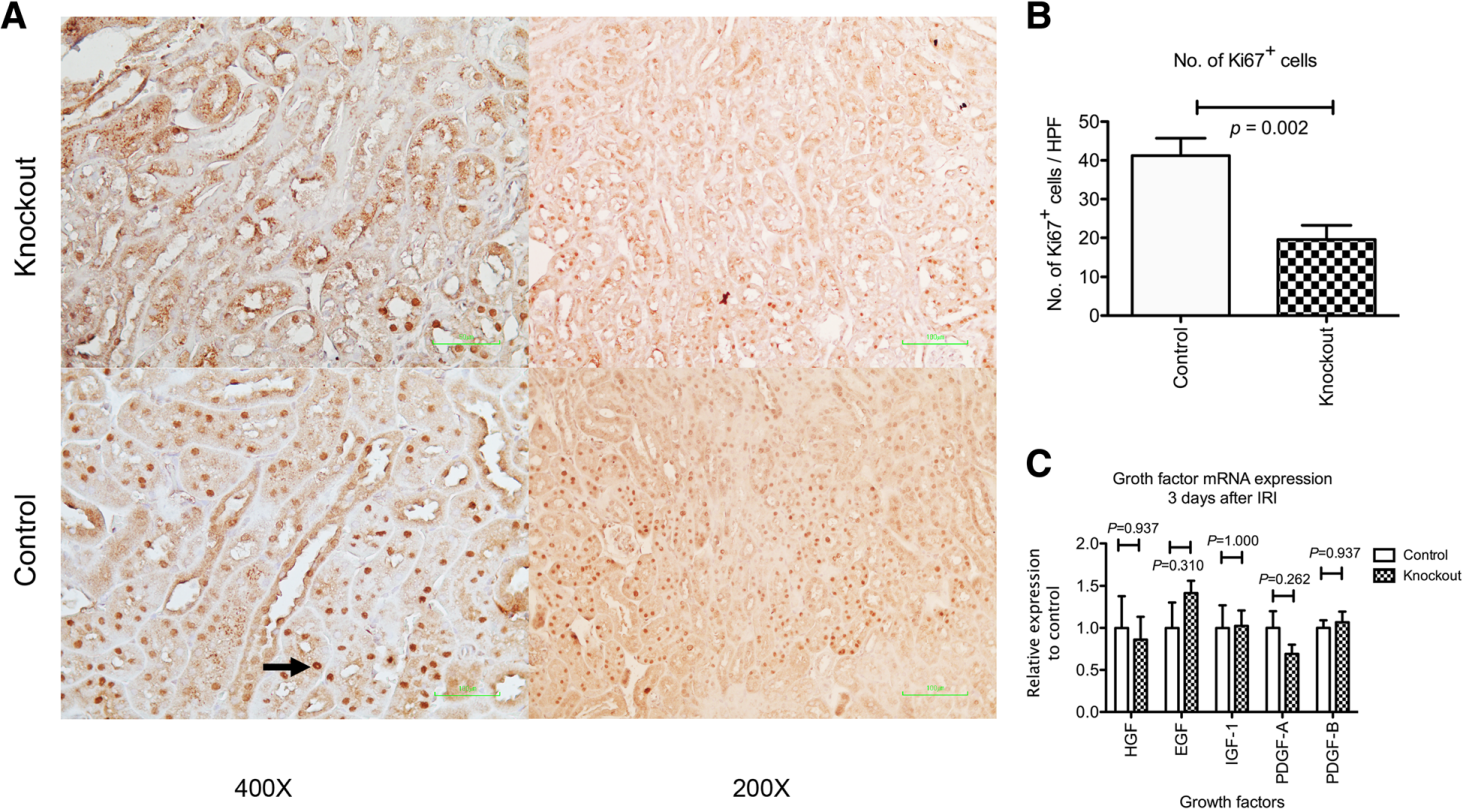
**a** & **b** Ki67 staining and semi-quantitative analysis of Ki67 positive tubular cells 3 days after IRI in the control and knockout mice. Fewer Ki67 positive cells were observed in the knockout mice. Arrow indicates Ki67 positive cells (*N* = 8 per group, *P* = 0.002) **c** Growth factor genes expression in the whole kidney. No difference of the gene expression of *HGF, EGF, IGF-1, PDGF-A* and *PDGF-B* was observed between the *Angpt1* knockout and control mice (*N* = 6 per group)

Subsequently, we examined tubular regeneration by Ki67 staining. Fewer Ki67 positive cells were observed in the *Angpt1* knockout mice 3 days after IRI (Fig. 5a, b). However, the gene expression of growth factors, including *HGF, EGF, IGF-1, PDGF-A* and *PDGF-B,* in the kidney 3 days after IRI did not differ between the control and knockout mice (Fig. 5c). Thus, the *Angpt1* deficiency did not change tubular regeneration by changing the expression of growth factors in the kidney.

### Angpt1 over-expression attenuated renal injury induced by IRI

**Fig. 6:**
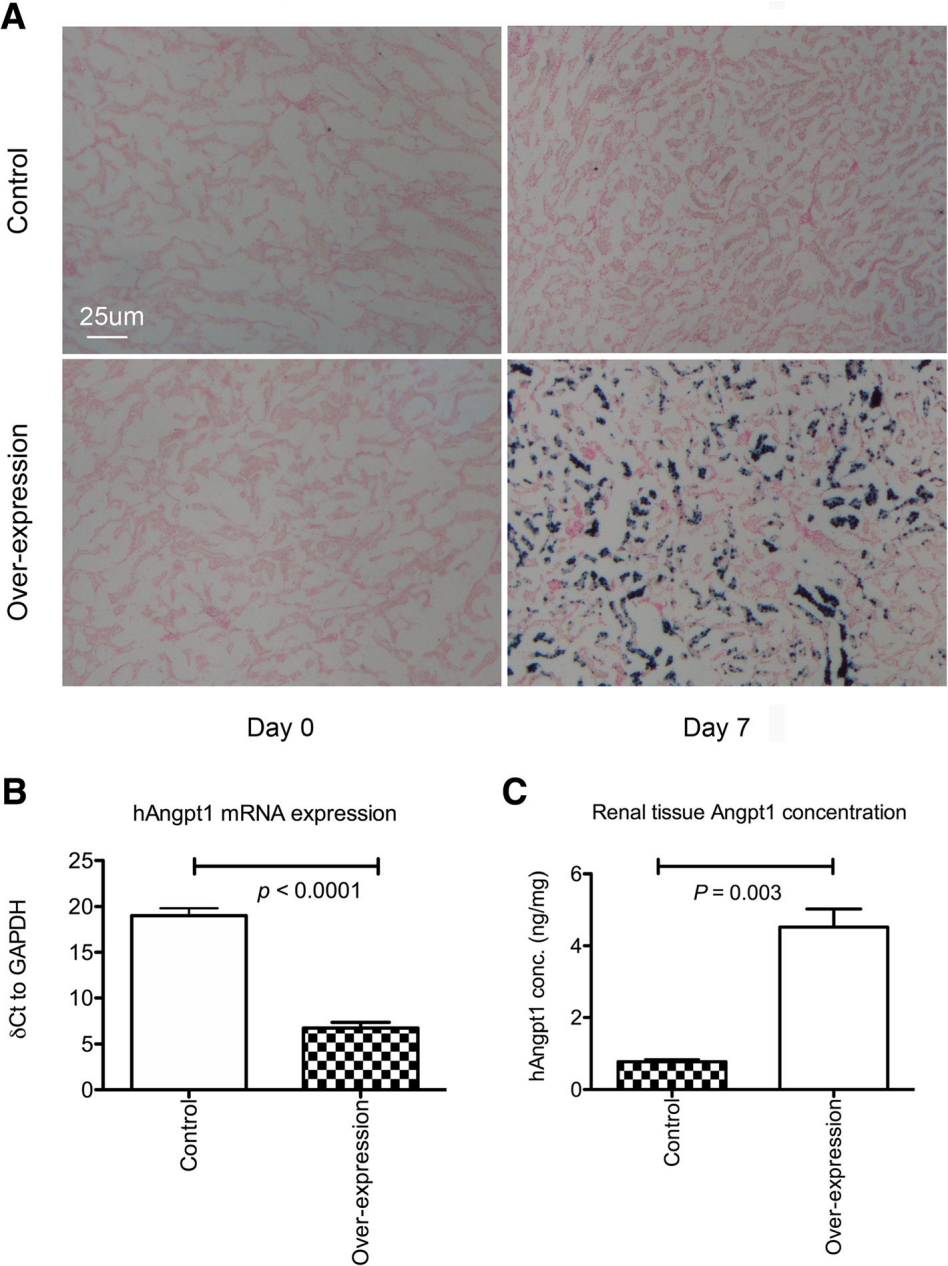
**a** X-gal staining in the *Angpt1* over-expression and control mice. Positive staining was observed in the tubular cells 7 days after the initiation of supplement of doxycycline in the *Angpt1* over-expression mice. The presence of positive staining indicates that the teteracycline responsive element promoter activity of *Angpt1.* The *β-galactosidase* activity in the over expression mice was activated. **b** ∆Ct (difference in the threshold cycle between *Angpt1* and *GAPDH*) of human *Angpt1* mRNA by quantitative RT-PCR analysis of control and human *Angpt1* over expression mice 1 day after IRI. The ∆Ct in the control mice was much higher than that in the over expression mice indicating significant over-expression of human Angpt1 mRNA was observed in the over-expression mice (*N* = 20 per group) **c** Renal tissue human Angpt1 concentration in over-expression and control mice. The over-expression mice had higher renal human Angpt1 concentration than control mice (*N* = 8 for each group)

**Fig. 7:**
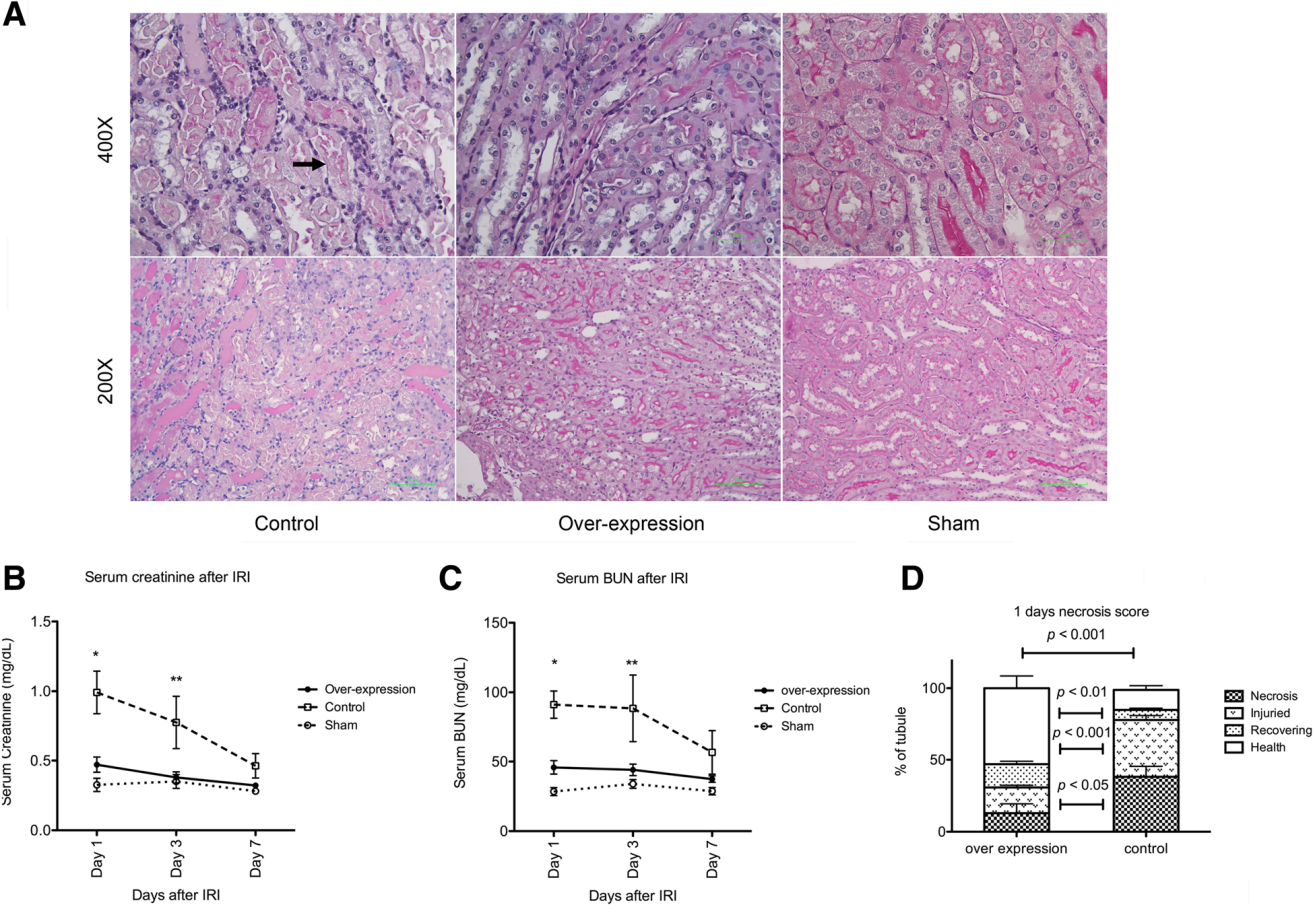
**a** PAS staining of renal tissue 1 day after IRI in control and over expression mice. The tubular necrosis was less prominent in the over expression mice 1 days after IRI. Arrow indicates necrotic tubule. **b** Serum creatinine level in the control and over expression mice 1, 3 and 7 days after IRI. The serum creatinine level was lower in the over expression mice 1 and 3 days after IRI. (*N* = 19 in the control group, *N* = 23 in the over-expression group, *N* = 5 in the sham mice, *P = 0.004, **P = 0.015) **c** Serum BUN level in the control and over mice expression 1, 3 and 7 days after IRI. The serum BUN level was lower in the over expression mice 1 and 3 days after IRI. (*N* = 19 in the control group, *N* = 23 in the over-expression group, *N* = 5 in the sham mice, *P < 0.001, **P = 0.016) **d** Semi-quantitative analysis of tubular necrosis 1 day after IRI in the control and over expression mice. The tubular necrosis score was less in the over expression mice. (*N* = 19 in the control group, *N* = 23 in the over-expression group, *N* = 5 in the sham group)

We investigated whether Angpt1 supplementation had a beneficial effect on renal IRI and examined its effect using inducible *Angpt1* over-expression mice. The administration doxycycline significantly induced RtTA binding promoter activity in the renal tubular cells which was presented as the β-glactosidase activity on X-gal metabolism in *Angpt1* over expression mice (Fig. 6a). The renal mRNA expression of human *Angpt1* was significantly up-regulated after the induction with doxycycline, and the expression level remained high 7 days after ischemic reperfusion injury but was almost undetectable or only detected in the very high threshold cycle using quantitative PCR in the control mice (Fig. 6b). Similar higher human Angpt1 protein level in kidney tissue was also noted 1 day after IRI in the over-expression mice (Fig. 6c). The over-expression of *Angpt1* in the adulthood didn’t induce abnormal different daily activity or organ gross appearance in the sham mice. The renal function was also not changed during the whole experimental course and there was also no renal morphological change observed by light microscopy 1 day after sham operation (Fig. 7a, b, c). Based on the lower serum creatinine and BUN levels 1 and 3 days after ischemic reperfusion injury in the over-expression mice, the over-expression of *Angpt1* can significantly attenuate the renal injury induced by ischemic reperfusion injury (Fig. 7b and c). The degree of renal tubular necrosis was also less severe in the over-expression group 1 day after ischemic reperfusion injury (Fig. 7a and d). Thus, the over-expression of Angpt1 enhances tubular cell survival during the early phase of stress induced by ischemic reperfusion renal injury. Similar results of less renal tubular necrosis were also seen 3 days after IRI (Additional file 3: Figure S3A and 3B).

### Angpt1 over-expression promoted endothelium survival after renal IRI

**Fig. 8:**
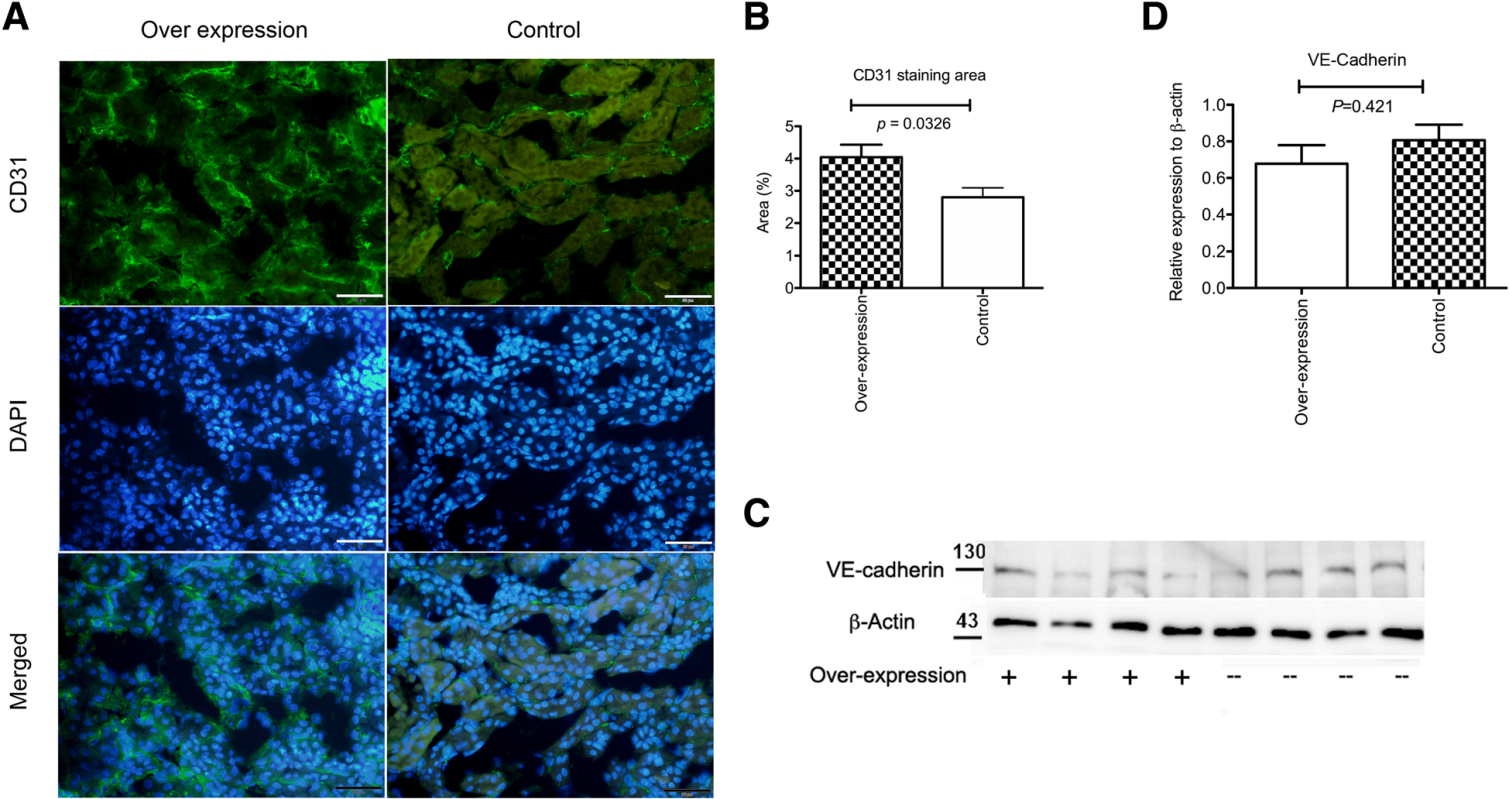
**a** & **b** CD31 endothelial staining and semi-quantitative analysis of the CD31 positive area 1 days after IRI in the control and over expression mice. The CD31 staining was more prominent in the over expression mice. (*N* = 15 per group, *P* = 0.033) **c** & **d** VE-cadherin protein expression 1 days after IRI in the control and over expression mice. The protein expression of VE-cadherin did not differ between the control and over expression mice (*N* = 4 per group)

Angpt1 is known to promote endothelium survival by activating the Akt signalling pathway (Kim et al., 2000; Papapetropoulos et al., 2000). We investigated whether Angpt1 supplementation could attenuate the endothelium loss induced by IRI. The Angpt1 supplementation by the over-expression in the kidney attenuated the loss of endothelial CD31 density 1 day after IRI (Fig. 8a and b). Similar finding was also found in over-expression mice 3 days after IRI (Additional file 4: Figure S4). However, the Western blotting results did not show any protective effect of additional Angpt1 on the loss of VE-cadherin 1 day after IRI (Fig. 8c and d).

### Angpt1 over-expression increased both M1 and M2 inflammatory genes expression

**Fig. 9:**
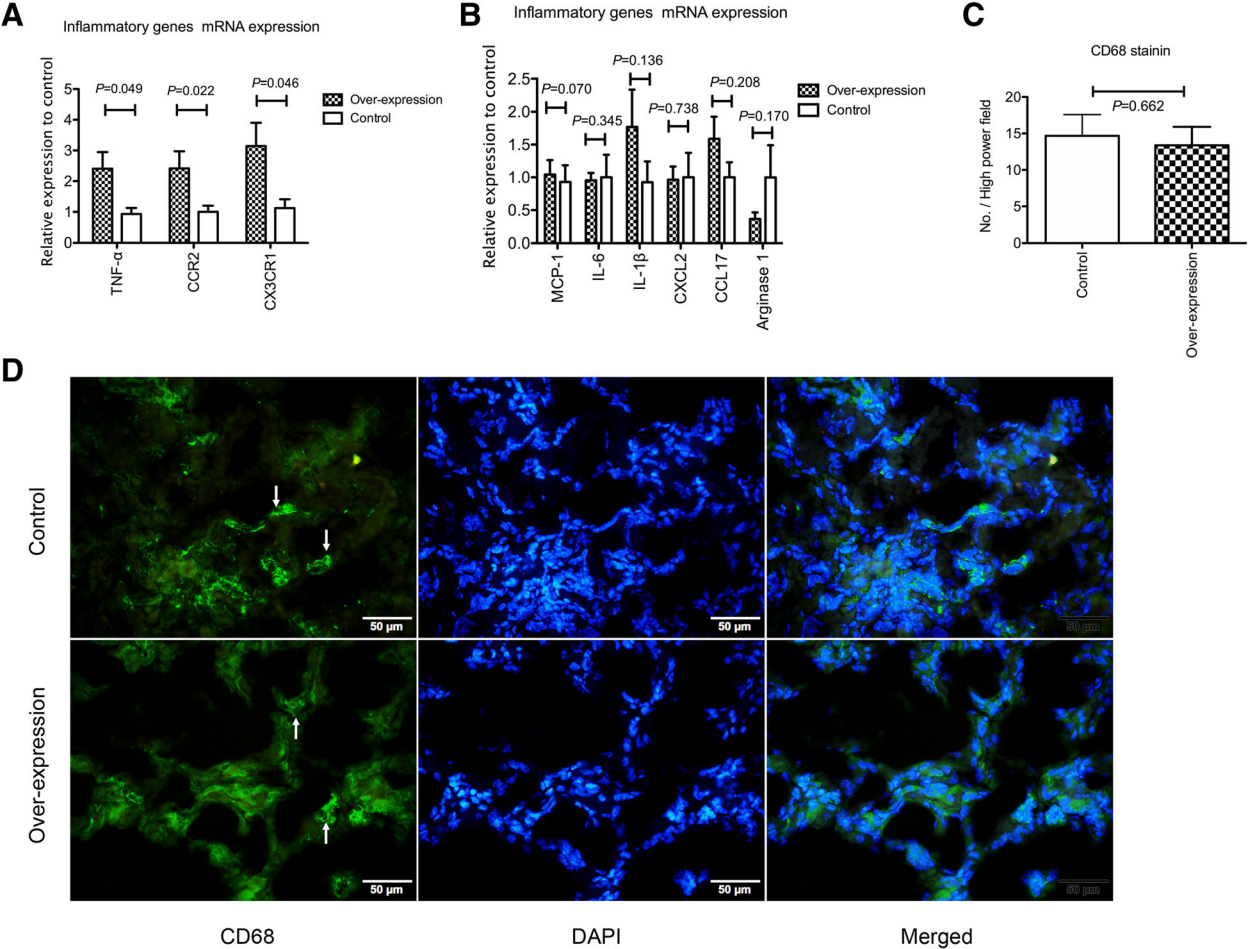
**a** & **b** Inflammatory genes expression 1 day after IRI in the whole kidney. The mRNA expression of *TNF-α, CCR2* and *CX3CR1* was higher in the over-expression than control mice. The gene expression of *MCP-1, IL-6, IL-1β, CXCL2, CCL17* and *arginase 1* did not differ between the over-expression and control mice. (*N* = 8 per group) **c** & **d** CD68 positive cells infiltration 1 day after IRI in the kidney. The infiltration of CD68 positive cells was not different between the over-expression and control mice. Arrow indicates CD68 positive cells (*N* = 6 per group)

The Angpt1 over-expression simultaneously stimulated the expression of the M1 type inflammatory genes *TNF-α and CCR2* and M2 type inflammatory genes *CXCR3* 1 day after ischemic reperfusion injury (Fig. 9a). No change was observed in the renal mRNA expression of *MCP-1*, *IL-6, IL-1β, CXCL2, CCL17*, or *Arginase 1* renal mRNA following the *Angpt1* over-expression (Fig. 9b). Although the inflammatory gene expression was higher in the over-expression mice, no difference was observed in the amount of macrophage infiltration 1 day after IRI between these two groups (Fig. 9c, d). Three days after IRI, there was no difference of M1 (TNF-α, IL-1β, MCP-1, IL-6 and CCR2) and M2 (CX3CR1, CCL-17, and Arg1) inflammatory genes between over-expression and control mice. Only the M1 inflammatory gene CXCL2 was higher in the control mice (Additional file 5: Figure S5).

### Angpt1 over-expression enhanced the renal expression of survival growth factors and attenuated tubular apoptosis

**Fig. 10:**
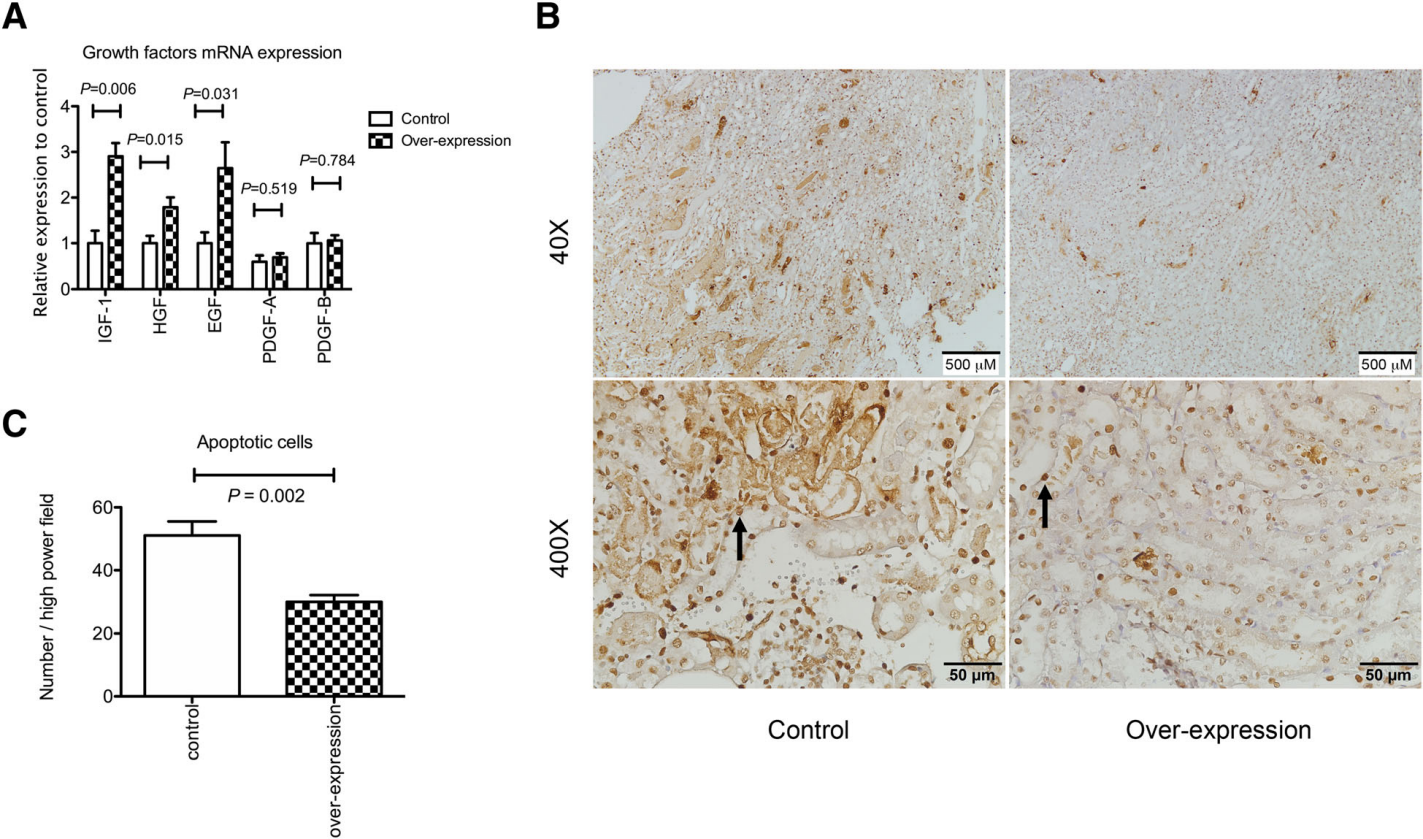
**a** Growth factor genes expression in the whole kidney. The mRNA expression of *EGF, HGF* and *IGF-1* was higher in the over-expression mice. There was no difference of *PDGF-A* and *PDGF-B* genes expression between the over-expression and control mice. (*N* = 8 per group) **b** Apoptotic tubular cells revealed by TUNNEL staining in the kidneys. There were more apoptotic tubular cells in the control than the over-expression mice. Arrow indicates apoptotic cell. **c** Semi-quantitative analysis of the apoptotic cells (*N* = 6 per group)

Tubular necrosis was attenuated by the *Angpt1* over-expression. Thus, certain factors enhanced the survival of tubular cells. The mRNA expression of *EGF, HGF* and *IGF-1* in the kidney tissue was up-regulated in the *Angpt1* over-expression mice. No difference was observed in *PDGF-A* and *PDGF-B* between the over-expression and control mice (Fig. 10a). The TUNNEL staining also demonstrated the tubular apoptosis was less prominent in the *Angpt1* over-expression mice (Fig. 10b and c). Thus, the less tubular apoptosis induced by over-expression of *Angpt1* was associated with up-regulation of some survival growth factors genes.

## Discussion

According to our results, Angpt1 deficiency impairs the processes of neoangiogenesis and recovery from renal IRI. In contrast, *Angpt1* over-expression attenuates the loss of the endothelium and renal tubular necrosis induced by IRI. Endothelial survival and regeneration were highly impacted by the alterations in *Angpt1* expression in our renal IRI model.

In normal mice, the renal *Angpt1* expression did not change one and three days after IRI, began to increase after seven days and was sustained 14 days after IRI. Thus, Angpt1 may play a role in the repair process during the later, but not early, phase of IRI. In our studies, the *Angpt1* knockout delayed the recovery from renal IRI three and seven days after injury. This finding is consistent with the finding of increased Angpt1 during the later phase of IRI. During the repair process after renal IRI, the development of neo-angiogenesis is important for establishing circulation in the renal tissue. Because Angpt1 mediates the remodelling and maturation of new vessels in angiogenesis (Thurston et al., 1999), the *Angpt1* knockout likely impairs the integrity of newly generated microvasculature during the later phase after IRI, which leads to sustained, increased capillary permeability and decreased renal blood flow. This hypothesis is supported by the reduced CD31 staining and decreased expression of VE-cadherin in the present study.

Furthermore, the renal function was better preserved and tubular necrosis was less severe in the over-expression mice 1 and 3 days after IRI. The immunostaining analysis demonstrated a reduced loss of CD31, indicating better endothelium survival after renal IRI. Angpt1 regulates endothelium survival via the PI3K/Akt signal transduction pathway (Kim et al., 2000; Papapetropoulos et al., 2000). Angpt1 also reinforces the endothelial tight junctions during endotoxemia (Hwang et al., 2009) and prevents VEGF-induced endothelial permeability (Gavard et al., 2008). Damage to renal vascular endothelial cells may contribute to the continued ischemia of renal tubular cells (Sutton et al., 2002). The restoration of the endothelial cell structure is associated with resolution in vascular tone and reversal of stasis, which improves the severity of ischemia. The high *Angpt1* level induced by over-expression appears to exert a renal protective effect by enhancing endothelium survival. This result is consistent with previous observations in other IRI organ damage, such as native or transplanted hearts (Lee et al., 2011; Syrjala et al., 2015). In addition, the expression of the growth factors *EGF, HGF* and *IGF-1* was augmented in the *Angpt1* over-expression mice. These growth factors could originate from surviving tubular cells, endothelial cell or other renal cells. Kobayashi et al. reported that Angpt1 could stimulate the endothelium to secrete HGF, which mediates smooth muscle cell recruitment (Kobayashi et al., 2006). Whether Angpt1 can stimulate the endothelium to exert protective paracrine function in renal IRI remains an unanswered question worthy of further exploration.

The inflammation status may pose a potential hazard to IRI renal damage mediated by Angpt1. Seok et al. reported that Angpt1 elicits pro-inflammatory responses in monocytes and differentiating macrophages (Seok et al., 2013). However, Angpt1 has been shown to reduce inflammation. Hegeman et al. reported that Angpt1 treatment reduced inflammation in ventilator-induced lung injury (Hegeman et al., 2010). The Angpt1/Tie2 signalling pathway inhibits the lipopolysaccharide-induced activation of RAW264.7 macrophage cells (Gu et al., 2010). In our *Angpt1* knockout mouse model, no difference was observed in the expression of pro-inflammatory genes, such as *TNF-α, IL1-β, MCP-1* and *IL-6*. Thus, the down-regulation of *Angpt1* does not appear to influence the inflammatory process during the first few days after renal IRI. In contrast, in the over-expression mouse model, the high levels of Angpt1 significantly activated the gene expression of *TNF-α*, *CCR2* and *CX3CR1*. In Li’s study, the number of macrophages increased in the mouse kidney one hour after reperfusion, peaked at 24 h and remained high for seven days. This infiltration was mediated by CCR2 and CX3CR1 (Li et al., 2008). However, in this study, the increase in *CCR2* and *CX3CR1* did not attract more macrophages to the renal tissue in the *Angpt1* over-expression mice. Although a higher inflammatory gene expression was observed in the *Angpt1* over-expression mice, the renal function remained preserved, and renal tubular necrosis was less prominent than that in the control mice. A more detailed examination of macrophage behaviour is required since certain macrophages also express the tie2 receptor, which may influence the M1/M2 phenotype switch. In a limb ischemia model, Angpt1-mediated Phd2 repression can switch Tie2 receptor-bearing macrophages to the pro-arteriogenic M2-like phenotype (Hamm et al., 2013). Tie2-expressing monocytes/macrophages may regulate revascularization in the ischemic limb (Patel et al., 2013). Tie2-bearing macrophages from patients with critical limb ischemia display greater proangiogenic activity than Tie2-negative monocytes in vitro (Patel et al., 2013). Tie2-expressing monocytes/macrophages support angiogenesis in tumours and remodelling tissues (Pucci et al., 2009; Coffelt et al., 2010; Capobianco et al., 2011; Mazzieri et al., 2011). The expression of both M1 inflammatory genes, such as *TNF-α/CCR2*, and M2 inflammatory genes, such as *CX3CR1*, was simultaneously up-regulated in the over-expression mice. Thus, the up-regulation of *Angpt1* likely prompts pro-inflammatory responses in conjunction with repair processes, including angiogenesis in renal IRI, eventually attenuating renal damage.

## Conclusion

*Angpt1* augmentation enhances endothelium survival during the early phase of renal IRI, and *Angpt1* deficiency impairs neo-angiogenesis and repair during the later phase of renal IRI. Thus, the down-regulation of Angpt1 in clinical conditions, such as sepsis, could be an important factor delaying recovery from AKI. Supplementation with high doses of Angpt1 could be beneficial in preventing renal damage and enhancing renal recovery from AKI.

## Additional files

Additional file 1: **Figure S1.** Renal function and proteinuria just before the induction of IRI in the *Angpt1* knockout and control mice. The serum creatinine level A), BUN level and C) urine albumin creatinine ratio was not different between the *Angpt1* knockout and control mice (*N* = 9 for each group). (TIF 321 kb)

Additional file 2: **Figure S2.** A) M1 inflammatory genes expression 7 days after IRI in the whole kidney. There was no difference of *TNF-α, IL-1β, MCP-1, IL-6, CCR2* and *CCL3* genes expression between the *Angpt1* knockout and control mice. (*N* = 8 per group) B) M2 inflammatory genes expression 7 days after IRI in the whole kidney. There was no difference of *CX3CR1, CCL17 and Arginase 1* genes expression between the *Angpt1* knockout and control mice (*N* = 8 per group). (TIF 301 kb)

Additional file 3: **Figure S3.** A) PAS staining of renal tissue 3 days after IRI in control and over-expression mice. The tubular necrosis was less prominent and healthy tubules were more in over-expression mice 3 days after IRI. B) Semi-quantitative analysis of tubular necrosis and tubular recovery 3 days after IRI in the control and over-expression mice. The tubular necrosis was less and more healthy tubules were noted in the over expression mice (*N* = 6 for each group). (TIF 34189 kb)

Additional file 4: **Figure S4.** A & B) CD31 endothelial staining and semi-quantitative analysis of the CD31 positive area 3 days after IRI in the control and over-expression mice. The CD31 staining was more prominent in the over expression mice (*N* = 9 per group, *P* = 0.038). (TIF 6861 kb)

Additional file 5: **Figure S5.** A) M1 inflammatory genes expression 3 days after IRI in the whole kidney. There was no difference of *TNF-α, IL-1β, MCP-1, IL-6, and CCR2* genes expression between the *Angpt1* over-expression and control mice. The mRNA expression of *CXCL2* was higher in the control mice (*N* = 8 per each group). M2 inflammatory genes 3 days after IRI in the kidney. There was no difference of *CX3CR1, CCL17 and Arginase 1* genes expression between the *Angpt1* over-expression and control mice (*N* = 8 per group). (TIF 262 kb)

## Abbreviations

AKI: Acute kidney injury
Angpt1: Angiopoietin 1
*Arg1*: *Arginase 1*
*CCL17*: *Chemokine (C-C motif) ligand 17*
*CCL3*: *Chemokine (C-C motif) ligand 3*
*CCR2*: C-C chemokine receptor type 2
*CX3CR1*: *CX3C chemokine receptor 1*
*CXCL2*: *Chemokine (C-X-C motif) ligand 2*
*EGF*: *Epidermal growth factor*
*HGF*: *Hepatocyte growth factor*
*IGF-1*: *Insulin like growth factor-1*
*IL-1β*: *Interleukin-1β*
*IL-6*: *Interleukin-6*
IRI: Ischemic reperfusion injury
*MCP-1*: *Monocyte chemoattractant protein-1*
*PDGF-A*: *Platelet derived growth factor-A*
*PDGF-B*: *Platelet derived growth factor-B*
Tie 2: Tyrosine kinase with Ig and EGF homology domains 2
*TNF-α*: Tumour necrosis-α
