# Supplementary figures and images for: Angiopoietin 1 influences ischemic reperfusion renal injury via modulating endothelium survival and regeneration
Source: Mol Med. 2019 Feb 13;25:5. doi: 10.1186/s10020-019-0072-7 (PMC6375134; doi:10.1186/s10020-019-0072-7)

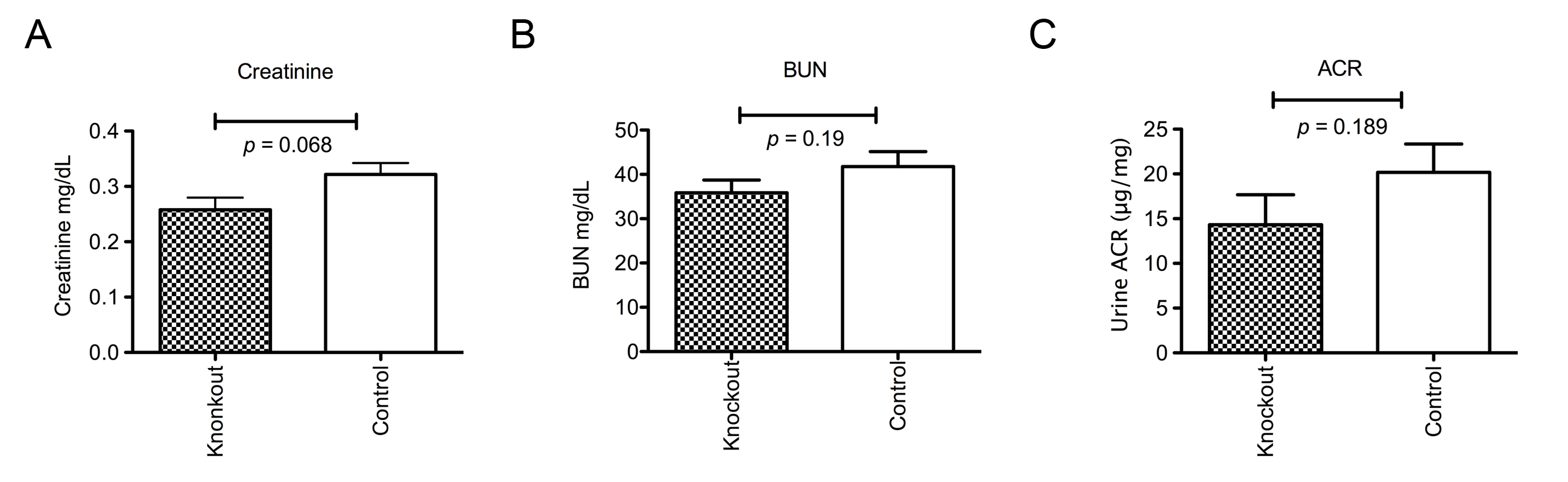

Supplement: Supplementary file 1 — Figure S1. Renal function and proteinuria just before the induction of IRI in the Angpt1 knockout and control mice. The serum creatinine level A), BUN level and C) urine albumin creatinine ratio was not different between the Angpt1 knockout and control mice (N = 9 for each group). (TIF 321 kb) [file 10020_2019_72_MOESM1_ESM.tif]

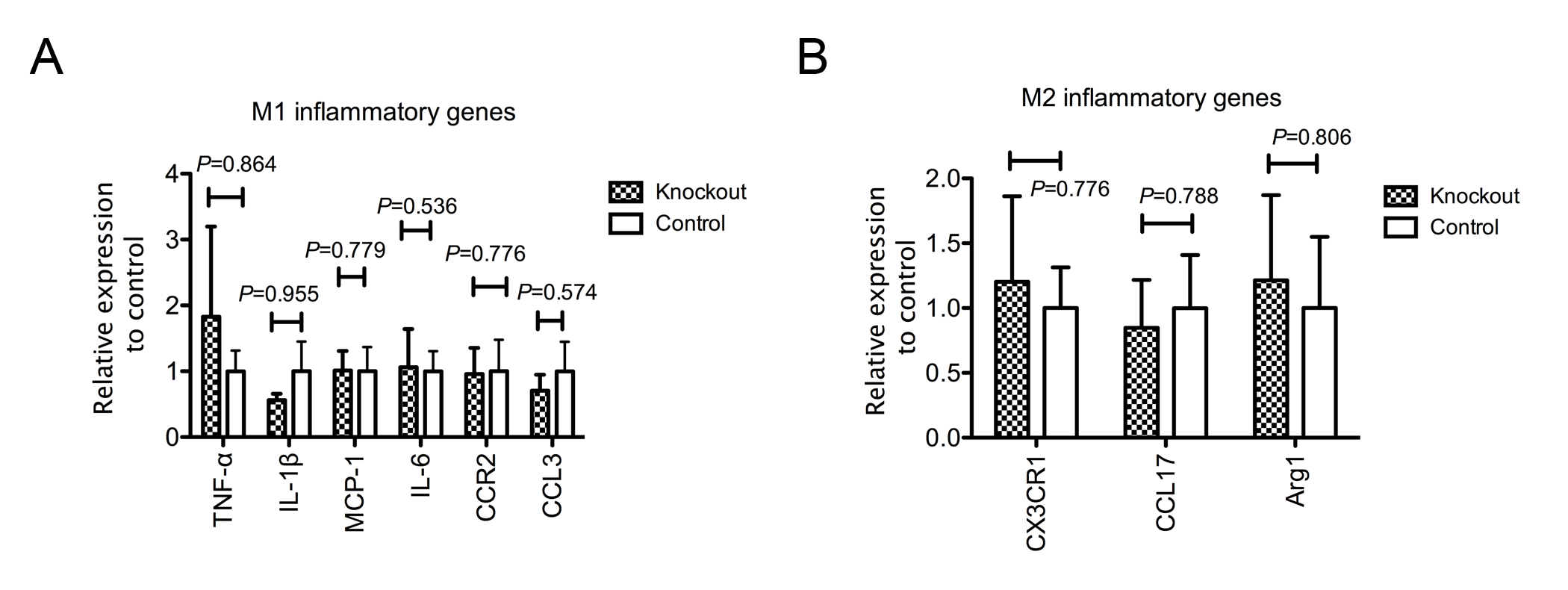

Supplement: Supplementary file 2 — Figure S2. A) M1 inflammatory genes expression 7 days after IRI in the whole kidney. There was no difference of TNF-α, IL-1β, MCP-1, IL-6, CCR2 and CCL3 genes expression between the Angpt1 knockout and control mice. (N = 8 per group) B) M2 inflammatory genes expression 7 days after IRI in the whole kidney. There was no difference of CX3CR1, CCL17 and Arginase 1 genes expression between the Angpt1 knockout and control mice (N = 8 per group). (TIF 301 kb) [file 10020_2019_72_MOESM2_ESM.tif]

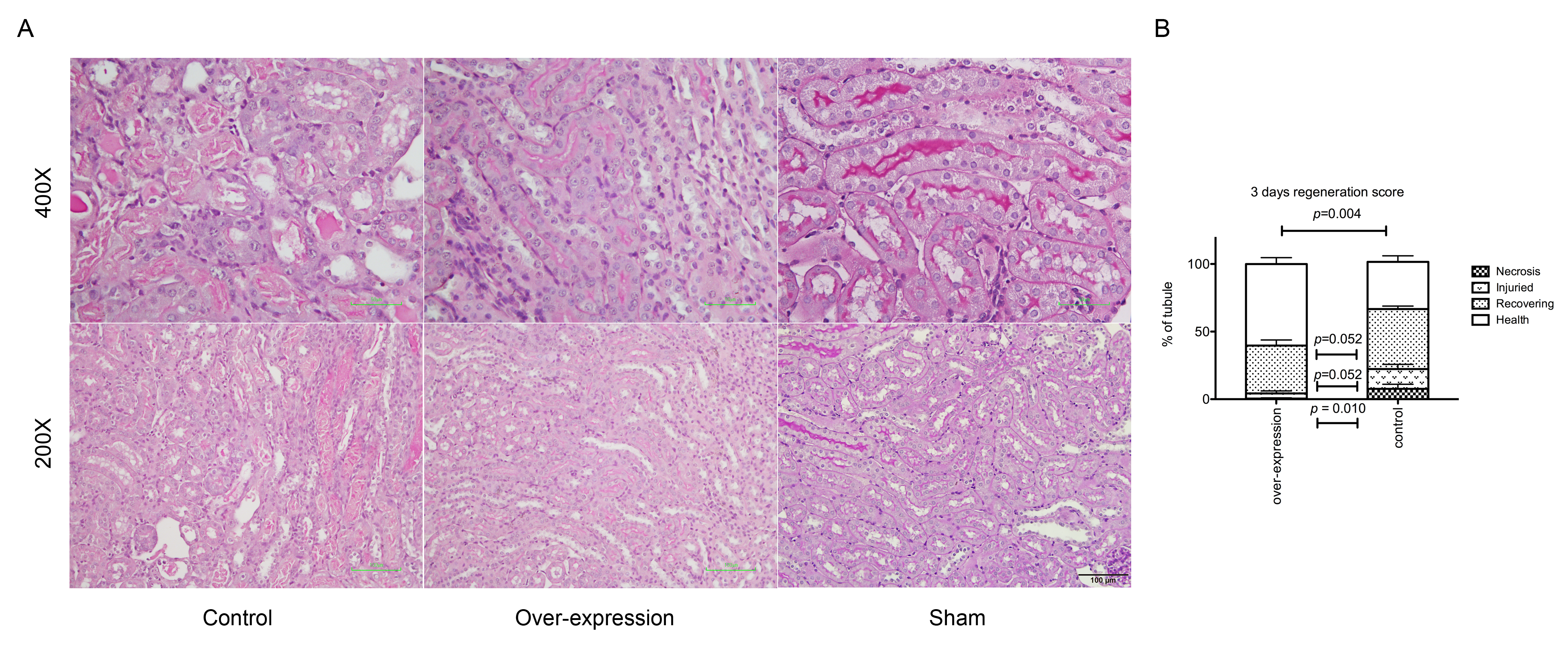

Supplement: Supplementary file 3 — Figure S3. A) PAS staining of renal tissue 3 days after IRI in control and over-expression mice. The tubular necrosis was less prominent and healthy tubules were more in over-expression mice 3 days after IRI. B) Semi-quantitative analysis of tubular necrosis and tubular recovery 3 days after IRI in the control and over-expression mice. The tubular necrosis was less and more healthy tubules were noted in the over expression mice (N = 6 for each group). (TIF 34189 kb) [file 10020_2019_72_MOESM3_ESM.tif]

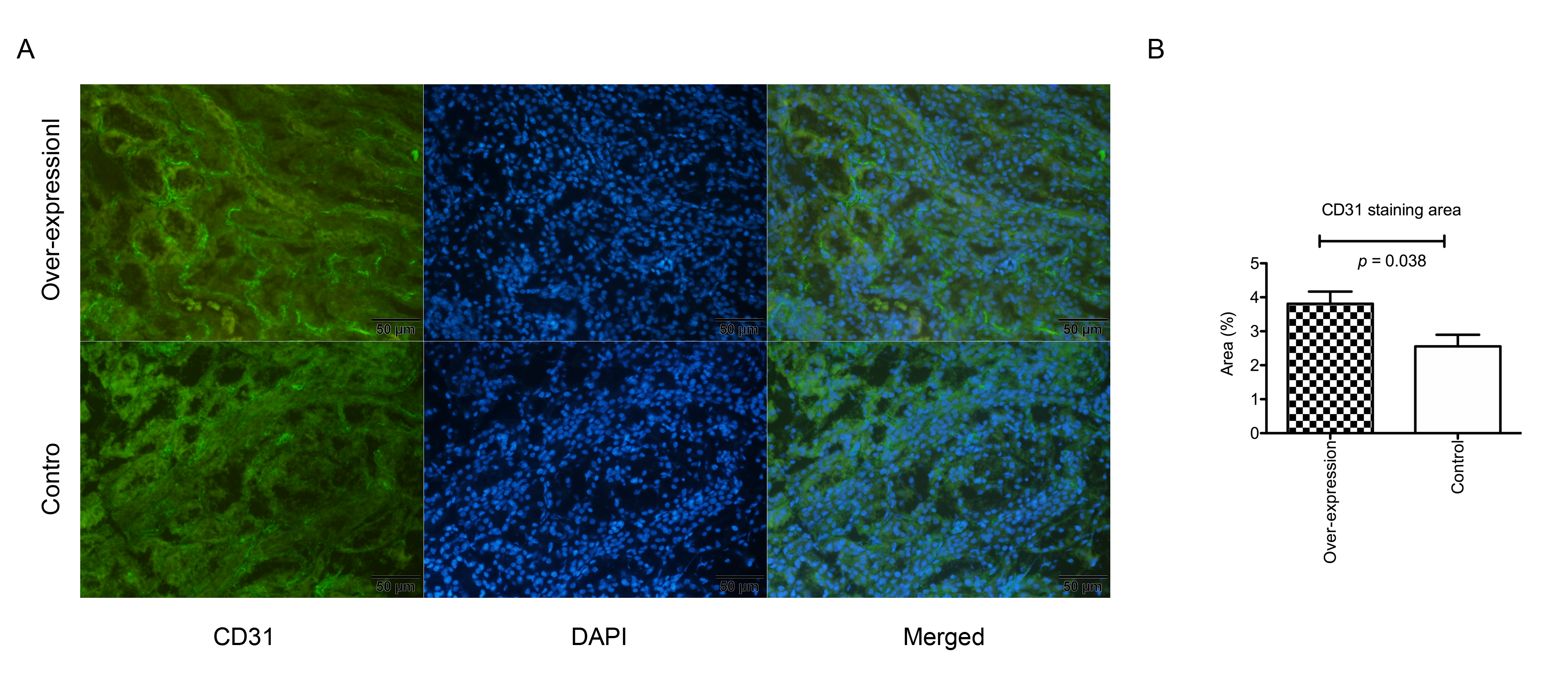

Supplement: Supplementary file 4 — Figure S4. A & B) CD31 endothelial staining and semi-quantitative analysis of the CD31 positive area 3 days after IRI in the control and over-expression mice. The CD31 staining was more prominent in the over expression mice (N = 9 per group, P = 0.038). (TIF 6861 kb) [file 10020_2019_72_MOESM4_ESM.tif]

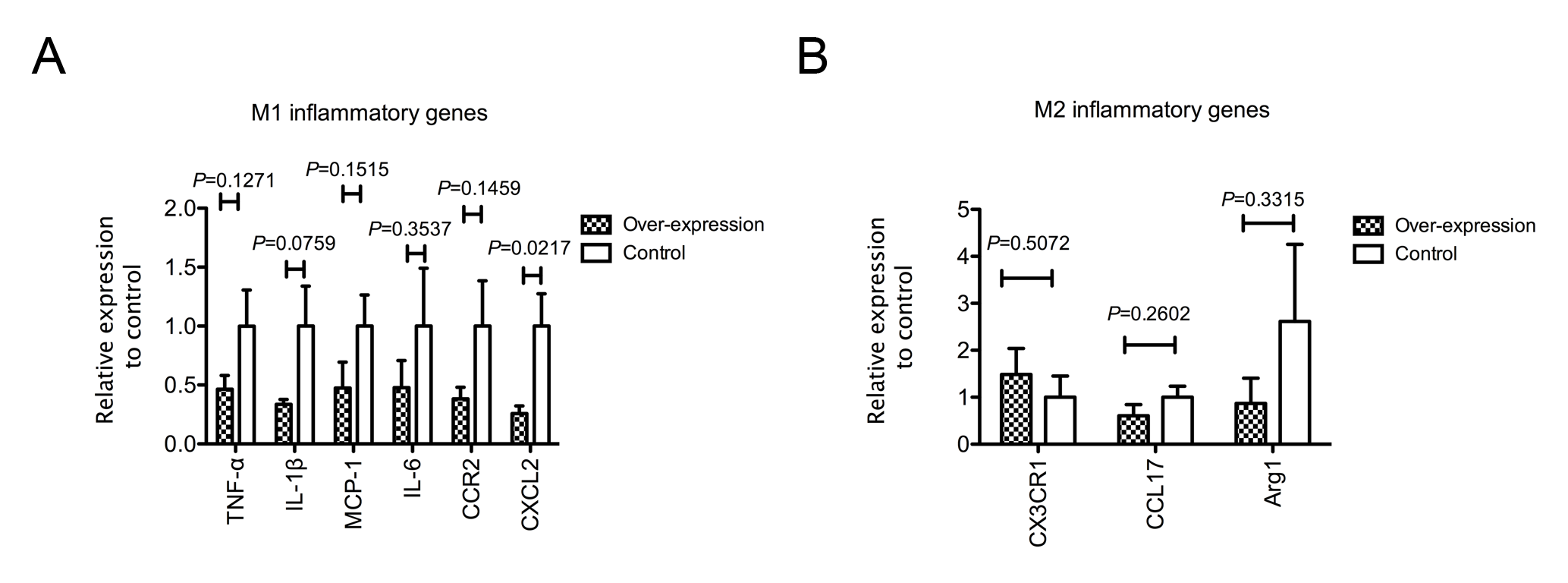

Supplement: Supplementary file 5 — Figure S5. A) M1 inflammatory genes expression 3 days after IRI in the whole kidney. There was no difference of TNF-α, IL-1β, MCP-1, IL-6, and CCR2 genes expression between the Angpt1 over-expression and control mice. The mRNA expression of CXCL2 was higher in the control mice (N = 8 per each group). M2 inflammatory genes 3 days after IRI in the kidney. There was no difference of CX3CR1, CCL17 and Arginase 1 genes expression between the Angpt1 over-expression and control mice (N = 8 per group). (TIF 262 kb) [file 10020_2019_72_MOESM5_ESM.tif]
